# Utility of late gadolinium enhancement cardiac MRI (LGE-CMRI) in troponin-positive chest pain with unobstructed coronary arteries

**DOI:** 10.1186/1532-429X-16-S1-P93

**Published:** 2014-01-16

**Authors:** Lucia Alvarez, R Brandon Stacey, Graham V Byrum, Gregory Hundley, Bharathi Upadhya

**Affiliations:** 1 Cardiology, Wake forest university Batist health, Winston Salem, North Carolina, USA

## Background

A surprising proportion of troponin- positive chest pain patients referred for contrast coronary angiography exhibit no or minor coronary arterial luminal narrowings at the time of presentation. We assessed whether the presence of late gadolinium enhancement (LGE) during cardiovascular magnetic resonance (CMR) forecasts adverse cardiovascular (CV) events in patient presenting with serum troponin elevations and chest pain but without obstructive coronary artery disease during contrast coronary angiography.

## Methods

CMR with LGE was performed in 77 consecutive patients who had been referred for CMR after presenting with new-onset chest pain, and an elevated serum troponin-I without coronary arterial luminal narrowings > 49% during contrast coronary angiography. The presence of major adverse cardiovascular events (MACE), defined as cardiac death, hospital admission for heart failure, or recurrent similar presentation were assessed by personnel blinded to all prior diagnostic testing.

## Results

Mean age was 52 ± 15 years; 51% were women. Out of 77 patients, 45 (58%) exhibited LGE [33% had patchy LGE consistent with myocarditis and 22% had subendocardial or full thickness LGE consistent with myocardial infarction (MI) ] and 33 (42%) had no LGE. Patients with LGE on CMRI were younger, more often male and had significant elevation of troponin-I elevations [8.7 (4-23.8 vs., 3.5(1.2-6.3); p = 0.001] and MB fraction of CPK isoenzymes [34.7(14-70) vs., 13(6.3-26.8); p < 0.001]. LVEF is lower with patients had no LGE [44 (36-53) vs., 51 (44-57); p = 0.007]. No other significant differences were observed with respect to CAD risk factors, EKG changes or severity of angiographic atherosclerosis. Patients with LGE had increased MACE compared to no LGE [22% vs., 6%, p = 0.104] over 24 months follow -up.

## Conclusions

LGE-CMRI is a useful tool for establishing the definitive evidence of MI and can make an important contribution to the long-term management strategy. Cardiac risk factors, EKG changes and angiographic atherosclerosis severity have no relationship to predict presence of LGE. The presence of LGE in these acute presentations may confer an adverse prognosis.

## Funding

Only departmental funds were needed.

